# Multiple origins and a narrow genepool characterise the African tea germplasm: concordant patterns revealed by nuclear and plastid DNA markers

**DOI:** 10.1038/s41598-017-04228-0

**Published:** 2017-06-22

**Authors:** Moses Cheloti Wambulwa, Muditha Kasun Meegahakumbura, Samson Kamunya, Alice Muchugi, Michael Möller, Jie Liu, Jian-Chu Xu, De-Zhu Li, Lian-Ming Gao

**Affiliations:** 1 Key Laboratory for Plant Diversity and Biogeography of East Asia, Kunming Institute of Botany, Chinese Academy of Sciences, Kunming, Yunnan 650201 China; 2 Germplasm Bank of Wild Species in Southwest China, Kunming Institute of Botany, Chinese Academy of Sciences, Kunming, Yunnan 650201 China; 3 College of Life Science, University of Chinese Academy of Sciences, Kunming, Yunnan 650201 China; 4 Genetic Resources Unit, World Agroforestry Centre, United Nations Avenue, P. O. Box, 30677 Nairobi, Kenya; 5 Genetic and Plant Breeding Division, Coconut Research Institute, Bandirippuwa Estate, 61150 Lunuwila, Sri Lanka; 6 Kenya Agricultural and Livestock Research Organization, Tea Research Institute (KALRO-TRI), Kericho, Kenya; 7 Department of Science, Royal Botanic Garden Edinburgh, Edinburgh, EH3 5LR Scotland UK; 8 Key Laboratory of Economic Plants and Biotechnology, Kunming Institute of Botany, Chinese Academy of Sciences, Kunming, Yunnan 650201 China; 9 World Agroforestry Centre, East and Central Asia Office, Kunming, Yunnan 650201 China

## Abstract

Despite the highly economic value of tea in Africa, its genetic and geographic origins remain largely unexplored. Here we address this by collecting 439 samples across 11 countries in Africa and Asia to investigate the origin and genepool composition of African tea based on 23 nuclear microsatellites loci (nSSRs) and three cpDNA intergenic spacer regions. Our results indicated that the African tea represents a potpourri originating from multiple introductions over time. The nSSR analysis revealed that the majority (79%) of tea accessions collected in Africa belong to Indian Assam tea which have likely originated from India and/or Sri Lanka. The patterns of nSSR variation also showed that Chinese Assam tea is genetically distinct from Indian Assam tea, and has rarely been used in African tea breeding efforts since only 4% of the African tea accessions possessed this genotype. We found a total of 22 cpDNA haplotypes, which grouped into three main geographic clades that were concordant with the distribution of microsatellite genotypes. Several private cpDNA haplotypes were identified in Chinese Assam tea in Southern Yunnan province of China. Therefore Chinese Assam tea will be important for the enrichment of African tea gene pools. Our results is a useful guide in future tea breeding programmes in Africa.

## Introduction

Tea is one of the most popular non-alcoholic beverages worldwide, which is consumed by approximately 70% of the world’s population for its refreshing taste, attractive aroma, therapeutic uses, and mildly stimulating properties^1^. In addition, tea plant extracts have shown potential in the control of malaria^2^, which is one of the biggest killer diseases in the tropics. The tea plant is also an important cash crop in more than 52 countries^3^, the main areas of tea production being Asia, Africa and South America. According to FAOSTAT^4^, Africa in particular, produced 699,057 metric tonnes of tea in 2014, which accounted for 13% of the global tea production and about 25% of the global black tea market. Africa’s tea export volume in 2015 was approximately 600,000 metric tonnes, earning the continent a total income of about US$1.5 billion. On relative terms, tea producing countries in Africa have been investing highly in tea research. For instance, in Kenya, at least one million US$ goes towards tea improvement annually^5^. However, the lack of information with regard to how Africa’s current tea genetic resources compare to the historically richer Asian tea germplasm might limit future progress in tea production in Africa. Africa’s tea breeding and germplasm conservation programmes stand to gain from a comparative genetic study between tea resources from Africa and Asia, as such a study is likely to expose germplasm utilisation gaps within Africa.

The tea plant [*Camellia sinensis* (L.) O. Kuntze] was the first documented tree crop in China, and has a long history of cultivation particularly in Yunnan and Sichuan provinces^6, 7^. It is undisputed that China is the area of origin of the tea plant. For instance, early writings from the year 59 BC during the Western Han Dynasty suggest that tea consumption was already a common practice^8, 9^. In addition, tea was one of the commodities that were traded via the Silk Road as early as 2 BC^10–13^. Early records on tea trade also show that tea was taken out of China to Japan in the 8^th^ century, to Europe in the 17^th^ century and later to India^14^. China and India are the two largest producers of tea globally and they are likely the sources of tea planting material for other countries. Early historical accounts of tea movement into Africa support two possible scenarios. The first possibility suggests that tea seed was introduced directly from China and then used to establish the first tea plantations in Africa. This scenario would represent a direct introduction of tea germplasm from China to Africa. The second possibility suggests that India and Sri Lanka were the ‘conduits’ through which the tea plant was introduced to Africa^14, 15^. According to this scenario, tea propagules from India and Sri Lanka were introduced between 1904 and 1912 to Africa. After its introduction by whichever route to Africa, the germplasm was probably disseminated among countries within the continent. To allow a targeted improvement of future breeding programmes in Africa, there is a need for an empirical determination of the origin of tea germplasm in Africa, as well as an identification of possible unrepresented genetic resources from the areas of origin.

Cultivated tea is mainly classified into two taxonomic varieties, *Camellia sinensis* var*. sinensis* with small, dark green leaves, and *C*. *sinensis* var. *assamica* with larger, lighter green leaves^16^. Some wild relatives of *C*. *sinensis* var. *assamica* occur in South China and neighbouring countries and several of these species are used as tea in China. Some of these wild species were found to have contributed in the historical domestication of tea and in modern tea breeding^17, 18^. Such genetic contribution is facilitated by the out-crossing nature of the tea plant^19^, and it is widely accepted that hybridization and introgression of wild relatives into the cultivated tea gene pool is likely to have occurred. For example, cpDNA haplotypes originating from Chinese Assam tea, *C*. *taliensis* and *C*. *irrawadiensis* have been detected in cultivated tea^18^.

A recent molecular study on Chinese and Indian tea germplasm demonstrated that cultivated tea can be separated into three genetically distinct groups: China tea (*C*. *sinensis* var*. sinensis*), and two types of Assam tea (*C*. *sinensis* var. *assamica*) that have been shown to constitute two independent lineages, one cultivated in Southwest Yunnan Province, China, and one in India^20^. Previous studies on the genetic architecture of African germplasm showed that the majority (at least 52%) of tea plants in cultivation in Africa are *C*. *sinensis* var. *assamica* that formed two genetically and geographically distinct groups in southern and eastern Africa respectively^18^. However, their links to the centres of origin of Assam tea in India and China had not been investigated.

Combined with maternally inherited plastid markers, nuclear simple sequence repeats (nSSRs) have revealed concordant genetic and geographic patterns in several studies, such as in olive^21^, *Arabidopsis* ^22^, algae^23^ and grape^24^, although discordant patterns have also been shown^25–28^. Although for tea there are relatively few studies published that utilized both nuclear and cpDNA markers to evaluate the genetic relationships across broad geographic areas [although see Wambulwa *et al*.^18^, for a more localized study], microsatellites have proven to be highly transferable between *Camellia* spp.^29^. CpDNA has been shown to be maternally inherited in the tea plant^30^, offering its potential utility for the evaluation of maternal lineages.

In the present study, we representatively sampled tea individuals from eight countries in Africa and three countries in Asia, namely China, India and Sri Lanka. We employed a combination of nuclear and chloroplast markers to genotype these sampled tea cultivars to: 1) determine the genetic and geographic origin of African tea germplasm, and 2) identify potential genetic resources missing from the African tea breeding programmes. The work reported here will be invaluable for the progress of future tea breeding programs in Africa, with the added value to feed baseline data into germplasm conservation initiatives of this important tree crop.

## Results

### nSSR data

**Figure 1:**
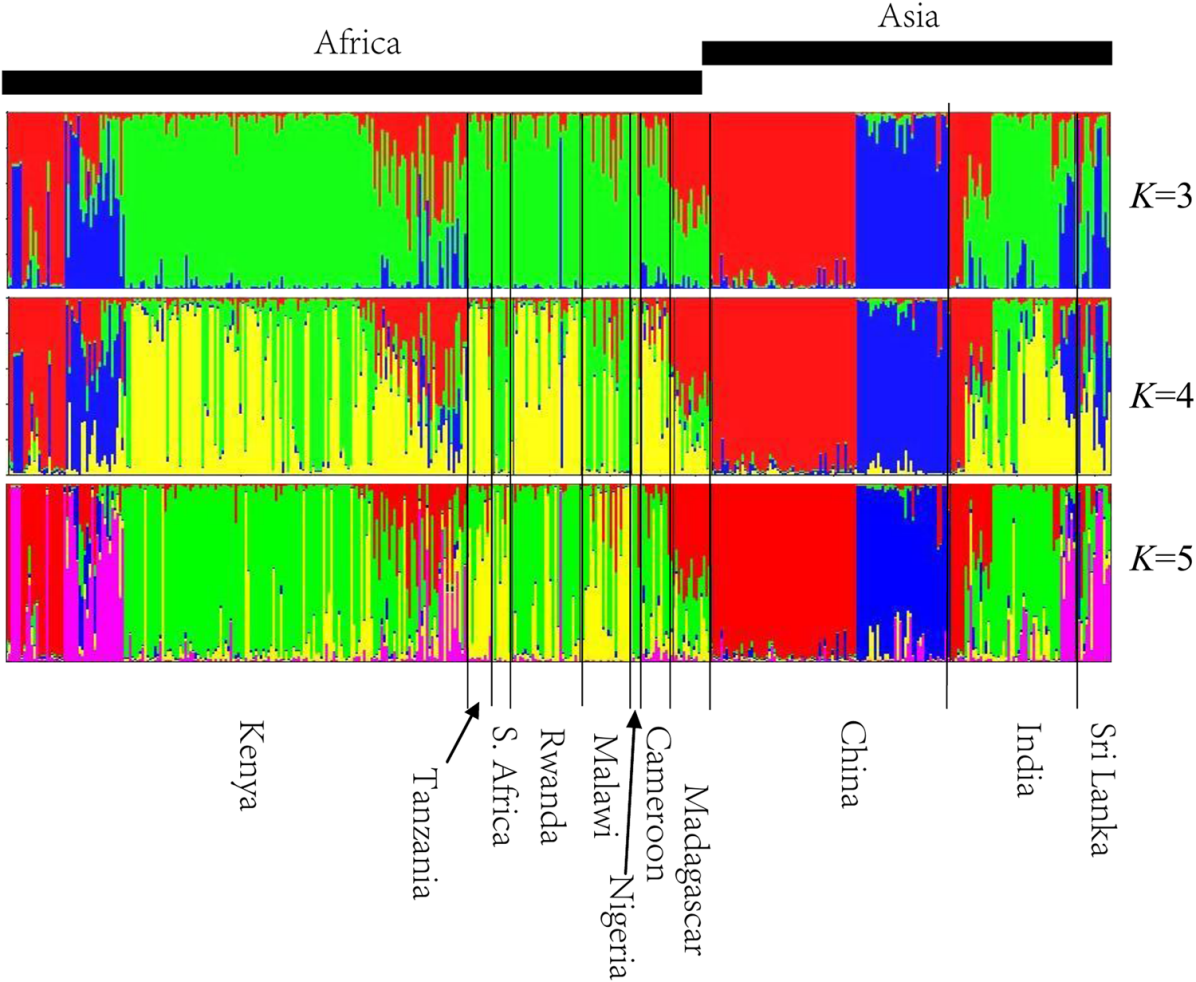
Bayesian assignment of probabilities for 439 tea individual from Africa and Asia. The inferred genetic groups are distinguished by different colors. The black lines delimit accessions from different countries. Each accession is indicated by a vertical bar, and the length of each colored section in each vertical bar represents the posterior probability that a given accession belongs to a particular genetic group.

Results of the Bayesian clustering are shown in Fig. 1. Based on the *L*(*K*) method, the optimal number of genetic clusters was found to be 3 (Fig. S1). At *K* = 3, the three genetic groups corresponded well with the three tea types recently identified in China and India: China tea, Chinese Assam tea and Indian Assam tea^20^. Most of the African accessions showed the same genetic background as the Indian accessions of Indian Assam tea (green) (Fig. 1; Fig. S2). The accessions from Madagascar and a small proportion of Kenya accessions grouped together with Asian samples of China tea (red). The Chinese Assam tea accessions were assigned to a distinct genetic group (blue) and several Kenyan and Sri Lankan accessions were of this type. At *K* = 4, a new genetic group (yellow) emerged from the Indian Assam tea group which was present in all sampled countries where this tea type occurred. At *K* = 5, a fifth genetic group was formed (pink), which included accessions from Kenya, India and Sri Lanka containing genetic composition of the Chinese Assam tea at *K* = 4 (Fig. 1).

**Figure 2:**
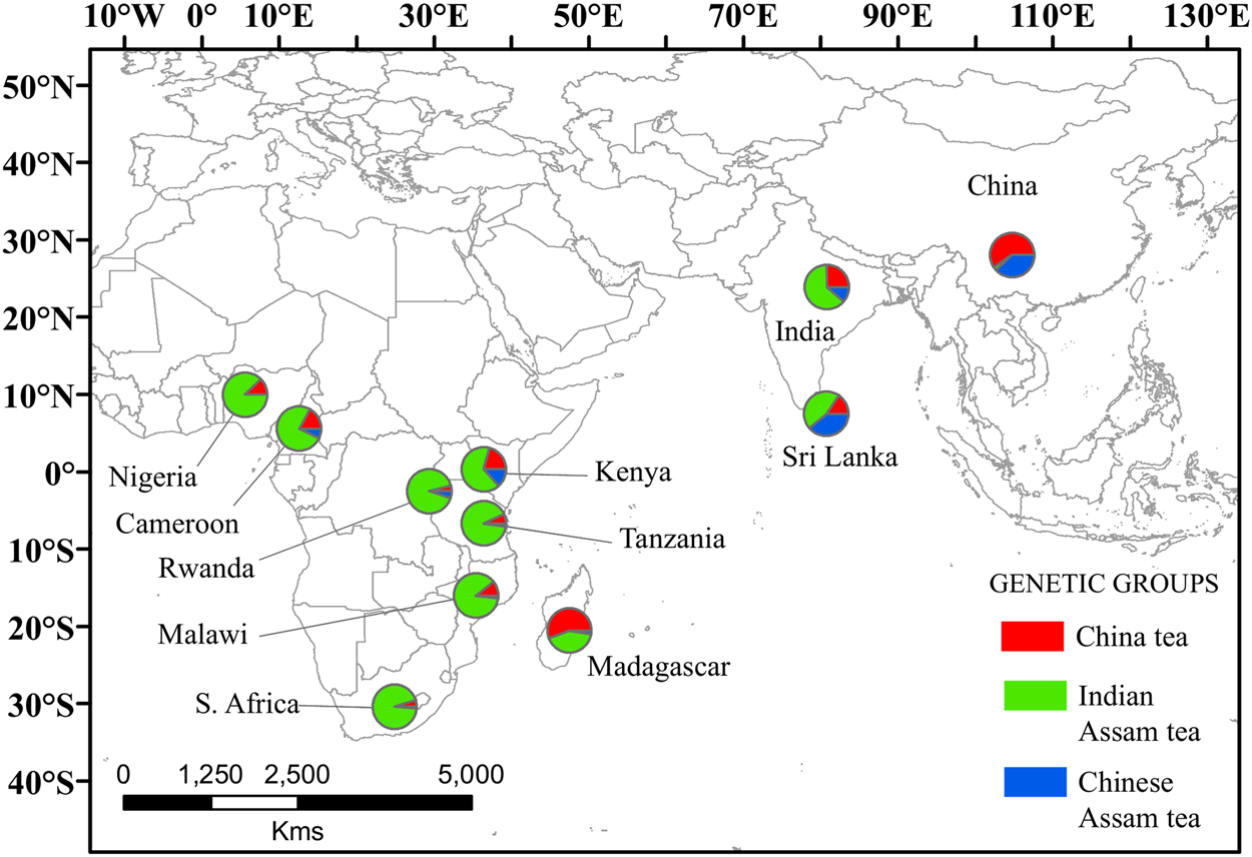
Geographic distribution of nSSR alleles. The colours correspond to the genetic clusters defined by STRUCTURE analysis at *K* = 3. The proportions of different groups in pie-charts are based on membership coefficients at *K* = 3. A shape file with genotype proportions in the different countries was generated in DIVA-GIS v7.5.0.0 (http://www.diva-gis.org/). The shape file was then used to generate the map in ArcGIS v10.2.2 (Environmental Systems Research Institute, Redlands, CA, USA) (http://www.esri.com/).

Geographic mapping of the nSSR genetic groups was performed based on posterior probabilities taken at *K* = 3, since this was performed in the *L*(*K*) method, and also in concordance with our previous study^20^. There was no geographic pattern discernible with the three tea types occurring in all African countries, the Indian Assam tea at similar high levels, except for Madagascar (Fig. 2). Across all samples, the nSSR genotypes of Indian Assam tea formed the most dominant group in Africa (79% of all tea accessions in Africa) and were mainly shared with India and Sri Lanka. The China tea gene pool accounted for 17% of African tea accessions and only occurred in Kenya outside China. Chinese Assam tea was predominantly distributed in China, with some accessions occurring also in Sri Lanka and India. It made up only 4% of the total tea accessions in Africa, with some accessions appearing mainly in Kenya.

Results of the PCoA scatter plot were consistent with the Bayesian STRUCTURE analyses and the samples formed three distinct clusters (Fig. S3). There was no clear geographic/genetic structure within Africa, and most African accessions grouped together with Indian Assam tea in one large cluster, with the Kenyan samples extending into the second cluster. This cluster comprised all China tea accessions from China, and India. The third cluster comprised mainly Chinese Assam tea individuals. According to these PCoA results, the Kenyan germplasm had the widest genetic base. A few accessions from Kenya, Rwanda and Sri Lanka appeared to have a close genetic relationship with Chinese Assam tea samples. In the neighbour joining tree (Fig. S4), the Chinese Assam tea group was genetically isolated from the rest of the accessions, with a few Kenyan accessions being part of the cluster near the base.

**Figure 3:**
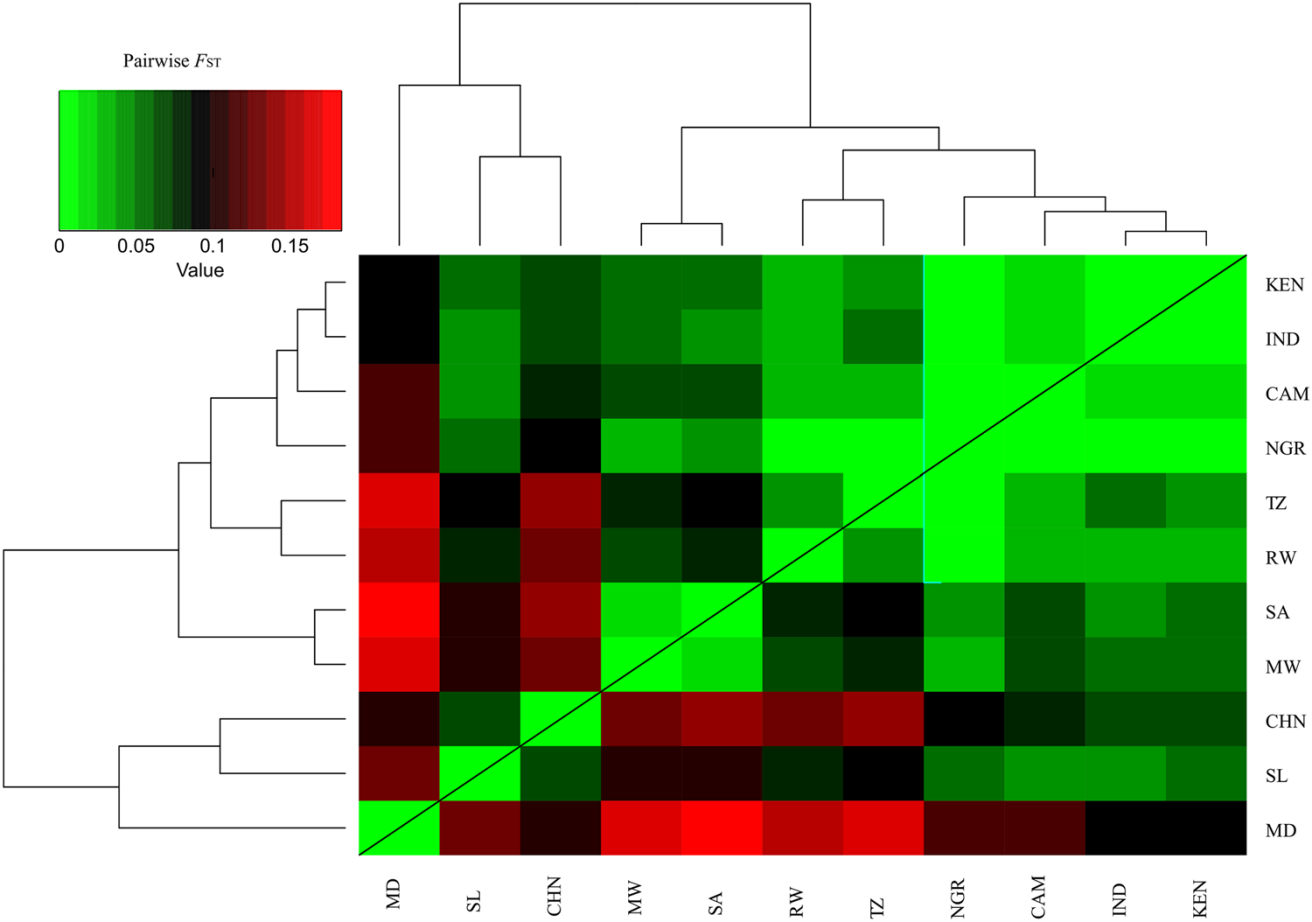
Heat map depicting pairwise *F*_ST_ estimates between countries. The map was generated by using the ‘heatmap.2’ function from the gplots package in R using pairwise *F*_ST_ matrix calculated in StAMPP. Populations (countries) are across the X- and Y-axis, with each square indicating the *F*_ST_ estimate between the two respective individuals. The magnitude of the genetic differentiation is indicated by the colour range. MD, Madagascar; SL, Sri Lanka; CHN, China; MW, Malawi; SA, South Africa; RW, Rwanda; TZ, Tanzania; NGR, Nigeria; CAM, Cameroon; IND, India; KEN, Kenya.

Partitioning of molecular variation at country level based on AMOVA (Table 1) revealed that 6.33% of the total variation was among countries, while 84.47% was distributed among individuals. The rest of the variation (9.20%) was distributed among individuals within countries. The highest pairwise *F*_ST_ value was observed between South Africa and Madagascar (0.18339) while the lowest (0.000) was found between Nigeria *vs* Kenya, Nigeria *vs* Tanzania, Nigeria *vs* Rwanda, Nigeria *vs* Cameroon and Nigeria *vs* India (Table S1; Fig. 3). We also noted generally high *F*_ST_ estimates between China and most African countries. There was no significant relationship between genetic differentiation and geographic distance (Fig. S5).

**Table 1:** Analysis of molecular variance (AMOVA) for tea individuals from all the 11 countries.

Source of variation	d.f.	Sum of squares	Variance components	% of variation
Among countries	10	472.515	0.57144	6.33
Among individuals	439	3349.5	7.62984	84.47
Total	449	3822.015	8.2013

### cpDNA sequence data

The alignment of the three sequenced cpDNA regions (*ndhF-rpl32*, *trnSGG-trnSr* and *trnSf1-trnGGG*) was 655 bp, 640 bp and 577 bp long, respectively. The combined 1872 bp long alignment matrix comprised 143 individuals, and included 34 single nucleotide substitutions and 23 indels of 1 to 3 base pair in length. The average number of nucleotide substitutions (D_xy_) for Africa *vs* China and Africa *vs* India were 0.00419 and 0.00299, respectively.

**Figure 4:**
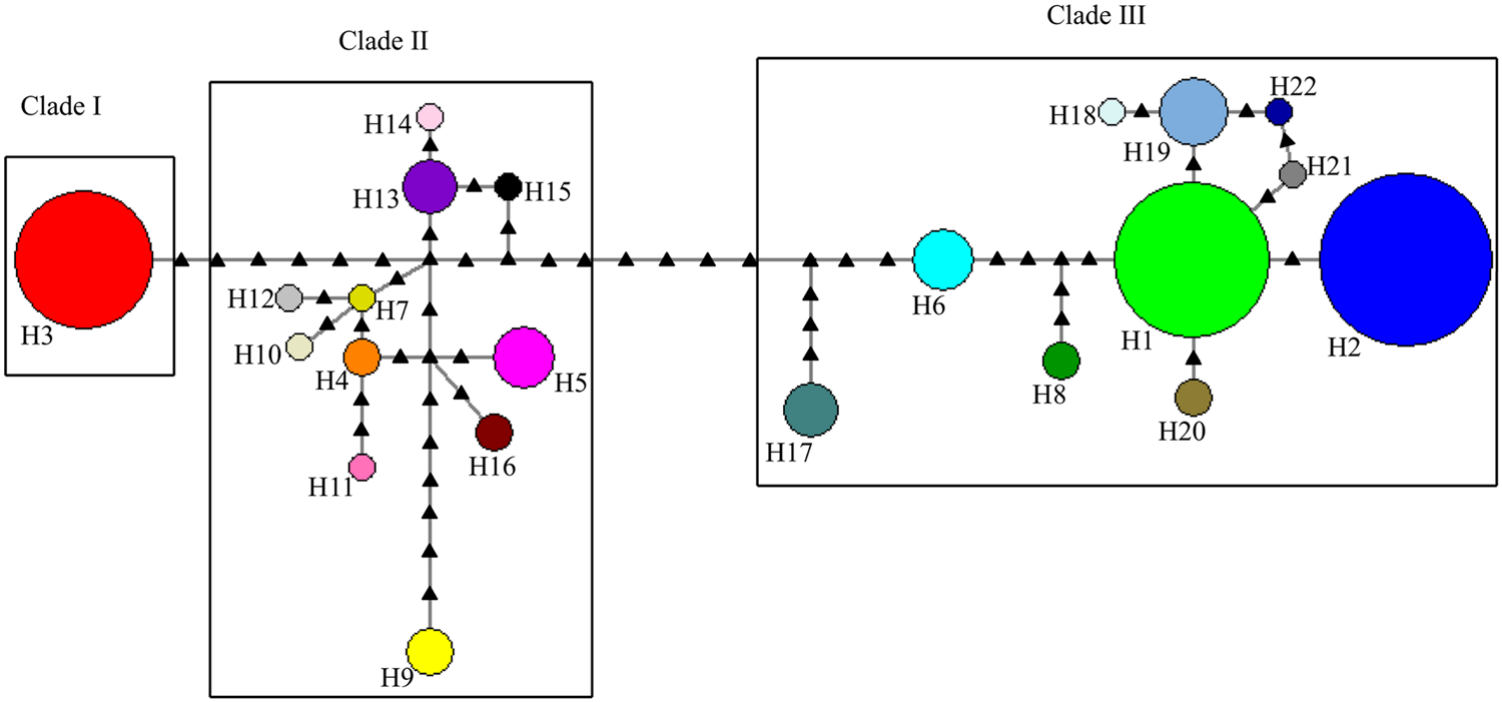
Median joining network of haplotypes of tea based on cpDNA sequence data. The size of the circles corresponds to the frequency of each haplotype whereas the small black triangles represent mutational steps.

We found a total of 22 haplotypes (H1 to H22) in the sequenced tea samples (Fig. 4; Table S2), with H1, H2 and H3 being the three major haplotypes as they were present in approximately 68% of the 143 individuals analysed. According to the haplotype network, the 22 haplotypes could be separated into three main clades, I, II & III, with six mutation events separating both, clade I and II, and clade II and III. Clade I contained only one haplotype (H3) comprising individuals of China tea from Kenya, Malawi, China and India. Clade II included 11 haplotypes (H4, H5, H7, H9, H10, H11, H12, H13, H14, H15 and H16), most of which represented Chinese Assam tea from Southern Yunnan, China (Fig. S6). The remaining 10 haplotypes formed Clade III (H1, H2, H6, H8, H17, H18, H19, H20, H21, H22), which mostly included accessions of Indian Assam tea from India and Africa and Chinese Assam tea from other parts of China. One accession of Chinese Assam tea (NNS4) was found to possess haplotype H1 (in Clade III) which was shared between India and Africa. We also noted that the haplotypes of Chinese Assam tea were all limited to China, with those originating from Southern Yunnan clustering closer to the China tea clade (Clade I) than those from other parts of China which clustered in Clade III.

Most of the haplotypes had limited geographical distributions (Fig. S6). For instance, six haplotypes were private in Africa: H4 and H5 (Kenya), H6 (Kenya & Rwanda), H7 (Cameroon), H8 (Kenya & Cameroon) and H9 (Madagascar). Eleven haplotypes (H10, H11, H13, H14, H15, H16, H18, H19, H20, H21 and H22) were private in China while two (H12 and H17) were private in India. The remaining three haplotypes were shared between the two continents. Clustering of the individuals in the neighbour joining tree was generally consistent with the results of the haplotype network (Fig. S7). We observed two main clusters, with one cluster corresponding to Clades I & II in the haplotype network. The other cluster corresponded to Clade III in the network.

The geographical distribution of the haplotypes was generally consistent with the distribution pattern of the nSSR alleles (Fig. S8). Haplotype H1 and H2 were shared among India, Sri Lanka and all African countries except Madagascar which shared its haplotype with China. Haplotype H3, which was associated with China tea accessions, was shared only between China, Kenya and Malawi.

## Discussion

East and Southeast Asia is the native home of the tea plant as well as comprises the most important centre of tea production worldwide. Especially China has a long history with of over 2000 years of tea domestication and cultivation. Following the domestication and economic utilization of the tea plant in China, it was introduced to other countries in Asia via the Silk Trade Road and Tea Horse Road^13^, and was thought to have subsequently been introduced to Africa by western missionaries and British imperialists^15, 31^. Our results confirmed this historical account as the majority of African and Asian tea accessions showed close genetic relationships, suggestive of a common origin (Fig. S3; Fig. S6). A recent study found that the African tea germplasm includes three genetic groups, China tea and two genetic groups of Assam tea in eastern and southern Africa^18^. We can now confirm that the two Assam tea groups in Africa derived from the Indian Assam tea lineage, while the African China tea originated initially from China.

A detailed knowledge of the history of crop development/domestication can help preventing genetic erosion, loss of ecotypes and landraces, and loss of habitat through land use change^32^. The relatively low amount of genetic variation between countries (Table 1) and the lack of geographic structure (Fig. S5) indicated that trans-national germplasm exchanges of tea germplasm have been a common practice among countries in Africa and Asia. Our recent study on the genetic structure of African tea hinted that the germplasm might have been derived from multiple geographic locations and the observed variation reflected genetic differences in the founding cultivars^18^. In the present study, we utilized patterns of genetic variation and Bayesian clustering to infer the origins of the tea plant in Africa, as a first attempt to provide an empirical account of the geographic origin of African tea plant. Consistent with some of the historical accounts of tea introduction into Africa, our results point to China and India as the geographic origins of African tea, with the dissemination involving the three previously reported genetic lineages of the tea plant^20^. Our Bayesian clustering and PCoA results showed that a large proportion of Africa’s tea germplasm is of the Indian Assam tea, which indicated that Assam tea in Africa possibly originated originally from India or indirectly from Sri Lanka. Our results here corroborated previous views that China is the origin of China tea which was introduced to Africa (mostly to Kenya) both directly (from Zhejiang and Guangdong provinces) and indirectly through Japan, India and Sri Lanka^15, 31, 33^. Meegahakumbura *et al*.^20^ also concluded that China tea originated from Southern China in a single domestication event, and was introduced from here to India in 1830s. Matheson and Bovill^15^ had alluded to the possibility that China tea hybrid seed had been introduced indirectly into Kenya through Sri Lanka in 1912. Our results further substantiated this possibility as a genetic group emerged in Kenya and Sri Lanka, which likely originated from artificial hybridisation and selection among a few individuals from India (Fig. 1). These China tea hybrid accessions were introduced to Kenya directly from Sri Lanka, but probably originated from India. More recently, some China tea accessions from Kenya, particularly the TRFK 800 series (Table S3), were introduced to Kenya through Material Transfer Agreements between China and Kenya^33^. To this end, our results support the hypotheses that the current African tea germplasm is the result of multiple origins and different introduction routes into Africa.

A striking observation from our nSSR analysis was the relatively low genetic differentiation and nucleotide substitutions (D_xy_) between India and African countries (Fig. 3; Table S1), and coupled with the high levels of genetic differentiation between China and Africa, might indicate that India has perhaps contributed more to the existing African tea germplasm. On the other hand, individuals from Madagascar were genetically distinct from samples collected in mainland Africa, which might indicate that tea samples in Madagascar have been introduced perhaps directly from China. There is no evidence for exchanges either way between Africa and Madagascar.

The geographic and genetic distribution of haplotypes also supported the idea that Africa’s tea germplasm originated from multiple sources with a large proportion originating from India. The two most frequent haplotypes (H1 and H2) were mainly shared between Africa, India and Sri Lanka, further confirming that India is the source of Africa’s Indian Assam tea. We also deduced that Africa’s China tea accessions descended from a single maternal lineage (H3), which originates initially from China. This finding supports the observation of Meegahakumbura *et al*.^20^ that domestication of China tea was a single domestication event followed by selection and development of cultivars from that particular single gene pool. Haplotypes H5 and H6 represented accessions of Cambod tea (*C*. *assamica* subsp. *lasiocalyx*), a group previously shown to be of hybrid origin between China tea and Assam tea^34^. The present study found that H5 and H6 occur in separate clades (clade II and III respectively), confirming the finding of Meegahakumbura *et al*.^20^ that the Cambod tea descended from at least two different maternal lineages. This observation underscores the importance of Cambod tea as a resource for tea improvement owing to its diverse genetic origins.

Intensive agricultural practices and overreliance on a narrow gene pool can reduce the genetic diversity of a crop species resulting in genetic homogeneity^35, 36^. Near clone-based plantations from a narrow gene pool will be very vulnerable not only to pests and diseases but also climate change. The genetically unique gene pools revealed in the present study are potential genetic resources for developing pest and climate smart agriculture^37–39^. We identified six private haplotypes in Africa (H4, H5, H6, H7, H8 and H9). The presence of private haplotypes within Africa was unexpected and may be due to persistent selection of agronomic traits that has resulted in a group of locally adapted individuals which are genetically distinct from the founder individuals^40^. However, given the fact that Africa’s tea cultivation history is relatively short, ca 100 years^15, 41^, the scenario of local selection and adaptation to local conditions seems unlikely to explain the distinct genetic divergences. Alternatively, the presence of private alleles and haplotypes in African may partly be due to the absence of wild relatives of the tea plant in our present analysis. It is possible that some tea gene pools in Africa share maternal lineages with wild closely related species of *Camellia* section *Thea* and have been used in breeding programmes to improve cultivars. For instance, haplotype H4 is present in *C*. *irrawadiensis* which was used to breed for tea leaf anthocyanins in Kenyan tea^18^. We also observed that H17, private in India, mainly comprised accessions of the ‘Tocklai Variety’ (TV) series from India. A BLAST analysis of the sequence of haplotype H17 returned a 100% match with *C*. *pubicosta* Merr. from North Vietnam^42^. It has been established that crop wild relatives (CWR) possess high genetic diversity that can contain traits beneficial in modern breeding programmes^43, 44^. The potential of utilising wild relatives of tea in germplasm improvement is immense; for example in some parts of China, leaves of *C*. *taliensis*, *C*. *grandibracteata*, *C*. *kwangsiensis*, *C*. *gymnogyna*, *C*. *crassicolumna*, *C*. *tachangensis*, *C*. *ptilophylla* and *C*. *irrawadiensis* from *C*. sect. *Thea* are used locally as source of a tea-like beverage^45, 46^.

The use of the recently delineated Chinese Assam tea^20^ was observed to be largely limited to China, although some of its nSSR alleles are present in Kenya and Sri Lanka, suggesting that some germplasm exchange and hybridization/introgression might have taken place. The geographic distribution of nSSR types also suggests that the Chinese Assam tea gene pool remains under-utilised in African tea breeding programmes. Previous studies have shown that Africa’s tea germplasm suffers from a narrow genetic base owing to the overutilization of a single parental resource for breeding^18, 34^. In light of this challenge, our study identified Chinese Assam tea as a valuable genetic resource for tea improvement in Africa. In addition, we noted that although China tea has been utilised in Africa to a considerable extent, there is still scope to increase its usage particularly in Rwanda, Tanzania, Malawi, South Africa and Nigeria in order to develop the climate-smart tea plantation.

## Conclusion

Our results demonstrated that African tea cultivars represent predominantly the Indian Assam tea, which was introduced from India and/or Sri Lanka. Furthermore, we found that the two recently delineated Assam tea gene pools in southern and eastern Africa were introduced directly from India or indirectly via Sri Lanka. China tea and Chinese Assam tea were introduced to Africa directly from China and possibly indirectly through other countries. Chinese Assam tea is currently seemingly underutilised in the breeding efforts in Africa and represents a potentially valuable genetic resource for the tea improvement in this country. Our findings will not only provide guidance for the genetic improvement of African tea cultivars but also facilitate efforts in germplasm conservation.

## Methods

### Sampling strategy

A total of 439 tea samples were collected from Africa and Asia. The sampled countries in Africa included Cameroon (13 samples), Kenya (183), Madagascar (15), Malawi (20), Nigeria (4), Rwanda (27), South Africa (8), and Tanzania (10). To ensure representation from all three possible centres of tea domestication in Asia^20^, 159 of the 439 individuals screened in this study were sampled throughout the tea growing areas in China (95), India (50) and Sri Lanka (14) (Table S3). Most of the samples in our collection belong to the two classical varieties of cultivated tea, *Camellia sinensis* var. *sinensis* and *C*. *sinensis* var. *assamica* representing the three known tea lineages defined in our recent study^20^: China tea, Chinese Assam tea, and Indian Assam tea and hybrids between China tea and Indian Assam tea. The sample set also included several accessions of Cambod tea (*C*. *assamica* subsp. *lasiocalyx*) and one cultivar of *C*. *irrawadiensis* (TRFK 91/1) from Kenya, which is an important genetic resource of tea breeding in East Africa.

### DNA extraction and nSSR genotyping

Genomic DNA extractions and nSSR genotyping followed the protocols outlined in Wambulwa *et al*.^34^. Twenty three polymorphic nuclear microsatellites previously used in Wambulwa *et al*.^34^ were also used to genotype all 439 individuals screened in this study. All forward primers were pre-labelled with fluorescent dyes. PCR products (1 μL) amplified by two or three different fluorescence dye-labelled primers were mixed with 9 μL of HI-DI™ formamide (Applied Biosystems, USA) and 0.5 μL of the GeneScan™ 500 LIZ Size Standard (Applied Biosystems, USA). The DNA fragments were denatured and size-fractionated using an ABI 3730xl DNA Sequencer (Applied Biosystems, USA). Subsequently, GeneMarker v2.2.0 (Applied Biosystems, USA) was used for estimation of allele size.

### Sequencing of cpDNA regions

In total, 143 individuals (84 African and 59 Asian samples) that were isolated for the nSSR study were also targeted for cpDNA sequencing, representing a broad sampling of tea genotypes across all 11 countries. PCR and sequencing of the three chloroplast intergenic spacers (*ndhF*-*rpl32*, *trnSGG*-*trnSr* and *trnSf1*-*trnGGG*) were performed according to Wambulwa *et al*.^18^. Forward and reverse chromatograms were assembled and visually checked independently by two investigators using Sequencher v5.0 (Gene Codes, Ann Arbor, MI, USA). The edited sequences of the three cpDNA regions were combined in SequenceMatrix v1.7.8^47^ and then aligned in Geneious v4.8.2 (http://www.geneious.com^48^) using the MUSCLE algorithm^49^ with a final rechecking of all variable sites in the original ABI trace files.

### Data analysis

For the nSRR data, the model-based Bayesian clustering approach in STRUCTURE v2.3.3^50^ was used to perform statistical inferences on the genetic structure of the tea samples. This approach assumes that the samples being analysed can be subdivided into theoretical panmictic clusters. The analysis was run 20 times for each value of *K* (the number of inferred groups) from 1 to 8 for 1,000,000 iterations after a burn-in period of 100,000 iterations. Structure Harvester^51^ was used to estimate the optimal value of *K* based on the L(*K*) method^52^. Pairwise genetic differentiation (*F*_ST_) was estimated in FSTAT v2.9.3^53^ while the heat map of the *F*_ST_ estimates between different countries was generated using the StAMPP R package^54^. To further analyse the genetic structure, a principal co-ordinate analysis (PCoA) was conducted in GenAlEx v6.5^55^ based on Nei’s unbiased genetic distances estimated between pairs of tea groups. Genetic relationships were analysed using the neighbor joining algorithm in Phylip v3.69^56^. We used Arlequin v3.11^57^ to conduct an analysis of molecular variance (AMOVA)^58^ to estimate the genetic variation that was assigned within and among countries.

For the cpDNA haplotypes, we used MEGA v6.0^59^ to construct a neighbor joining tree without an outgroup. Nucleotide substitutions per site between groups (D_xy_)^60^ and the number of haplotypes was calculated from aligned DNA sequences with DnaSP v5.10^61^. We estimated D_xy_ only for Africa *vs* China and Africa *vs* India (Sri Lanka was excluded from this analysis because we had only two sequences and this could potentially compromise the result due to a low sample size). Network v4.6.1.3 (Fluxus Technology Ltd.) was used to estimate the degree of relatedness among cpDNA haplotypes based on median joining algorithm. In the neighbor joining and network analysis, indels were treated as single mutational events.

The geographic distribution of the cpDNA haplotypes and nSSR genotypes were based on shape files generated in DIVA-GIS v7.5.0.0 (http://www.diva-gis.org/), imported to ArcGIS v10.2.2 (Environmental Systems Research Institute, Redlands, CA, USA) (http://www.esri.com/) using map projections based on the WGS 1984 World Geographic Coordinate Systems. The world map shape file was downloaded at http://www.diva-gis.org/. For purposes of clarity, the cpDNA haplotypes in southern Yunnan Province were mapped separately from those in other parts of China.

## Electronic supplementary material

supplementary material

Table S3
