# Supplementary material for: Multiple origins and a narrow genepool characterise the African tea germplasm: concordant patterns revealed by nuclear and plastid DNA markers
Source: Sci Rep. 2017 Jun 22;7:4053. doi: 10.1038/s41598-017-04228-0 (PMC5481375; doi:10.1038/s41598-017-04228-0)
Supplement: Supplementary file 1 — supplementary material [file 41598_2017_4228_MOESM1_ESM.pdf]

Multiple origins and a narrow genepool characterise the African tea germplasm: concordant patterns revealed by nuclear and plastid DNA markers

Moses Wambulwa, Muditha Kasun Meegahakumbura, Samson Kamunya, Alice Muchugi, Michael Möller, Jie Liu, Jianchu Xu, De-Zhu Li, Lian-Ming Gao

Table S1: Pairwise F_{ST} among the 11 countries. Countries were assumed to be populations.

	China	India	Sri Lanka	Kenya	Tanzania	Rwanda	Madagascar	Malawi	Cameroon	Nigeria
India	0.06915									
Sri Lanka	0.07101	0.04242								
Kenya	0.07118	0.01008	0.05266							
Tanzania	0.13876	0.05542	0.09738	0.04719						
Rwanda	0.12636	0.02737	0.07838	0.03626	0.03994					
Madagascar	0.10930	0.09329	0.12920	0.09440	0.16769	0.15128				
Malawi	0.13163	0.05649	0.10725	0.05832	0.08327	0.06586	0.16370			
Cameroon	0.08386	0.02171	0.04879	0.02380	0.03249	0.03041	0.11281	0.06916		
Nigeria	0.09025	0.00000	0.05306	0.00000	0.00000	0.00000	0.11393	0.03102	0.00000	
S. Africa	0.14538	0.04570	0.10485	0.05145	0.08576	0.07405	0.18339	0.01282	0.06799	0.03911

Table S2: Composition and distribution of the 22 cpDNA haplotypes

**Haplotype**	**Individuals**	**Country**
H1	EPK_TN14_3 'TRFK_73/3' 'TRFK_395/2' 'TRFK_6/8' 'TRIT_201/16' 'TRIT_201/44' 'TRIT_201/50' 'TRIT_201/73' 'TRIT_201/75' 'TRIT_201/82' '15/37' G539 'K/29' '8/9' '35/49' PMC_B1 'K/108' IB241 IR4 Var_68 Var_318 Var_143 CL_301 CL_303 'TRFK_303/577[+] 'TFRK_303/178' 'TFRK_303/216' TV9 TV25 'Singpo_1' 'TRI_4052' NNS4	Kenya, Tanzania, Rwanda, Nigeria, Cameroon, China, India, Sri Lanka
H2	'TRFK_430/90' 'TRFK_76/3' BBK_5 'TRFK_52/1' 'TRFK_829/3' 'TRFK_829/7' 'TRFK_831/1' '16/4_Mukumbani_BB_S '16/4_Mukumbani_SFS_ '16/4_Mukumbani_SFS_ '16/4_Shivatse' 'AHP_S15/10' IB79 'IB/108' Indian_hybrid_Seedli SFS_371 MT_12 PC_108 PC_165 PC_185 PC_117 Var_BB35 CL_168 CL_671 KTDA_B1 'MICHI__5/1/1/20' BBK_35 BBK_21 'TRFK_303/259' 'TRFK_430/52' 'TRFK_430/63' 'TRFK_375/5' 'TRFK_383/4' 'TRFK_371/3' 'TRI_4071' TV2 TV5 TV6 TV8 TV12 TV13 'AUGC_1'	Kenya, South Africa, Rwanda, Malawi, Nigeria, India, Sri Lanka
H3	TRFK_St_536 'TRFK_830/12' 'TRFK_830/15' CL_17 China_hybrid_seedling, Namroop 6, Huangdao Tea, Dabai Tea, 'Caicha_5, Huangshan Tea, 'Namroop_1' 'Namroop_8' 'Namroop_10' Guiyang 1, Guiyang 7, Guiyang 8, Wuyuan 2,	Kenya, Malawi, China,

	Longjing Tea 1, Caicha 1, Longjing Tea Yucha 1, Longjing Tea Yucha 3, Longjing Tea Yucha 6, Huaqiu 08, Mingshan 131, Liuanguapian	India
H4	'TRFK_306/4' 'TRFK_91/1'	Kenya
H5	'TRFK_657/1' 'TRFK_824/1' 'TRFK_301/1' 'TRFK_301/2' 'TRFK_301/3'	Kenya
H6	'TRFK_713/1' '12/56' 'TRFK_301/4' 'TRFK_301/5' 'TRFK_301/6'	Kenya
H7	CL_1932	Cameroon
H8	'CL_H81/22' 'STC_L6__38/8_'	Kenya, Cameroon
H9	MD03 MD04 MD15	Madagascar
H10	NL2	China (Southern Yunnan)
H11	XD13	China (Southern Yunnan)
H12	TV 21	India
H13	DA3 MH2 MQ7 YT15	China (Southern Yunnan)
H14	Baihao	China (Southern Yunnan)
H15	JX11	China (Southern Yunnan)
H16	JM13 JM15	China (Southern Yunnan)
H17	TV15 TV23 TV29 'Powei_4'	India

H18	ZD6	China
H19	GM9 MK7 LBZ11 BW1 WQ6 FS8	China
H20	JL9 LME7	China
H21	Zijuan Tea	China
H22	Bangdong Assam Tea	China

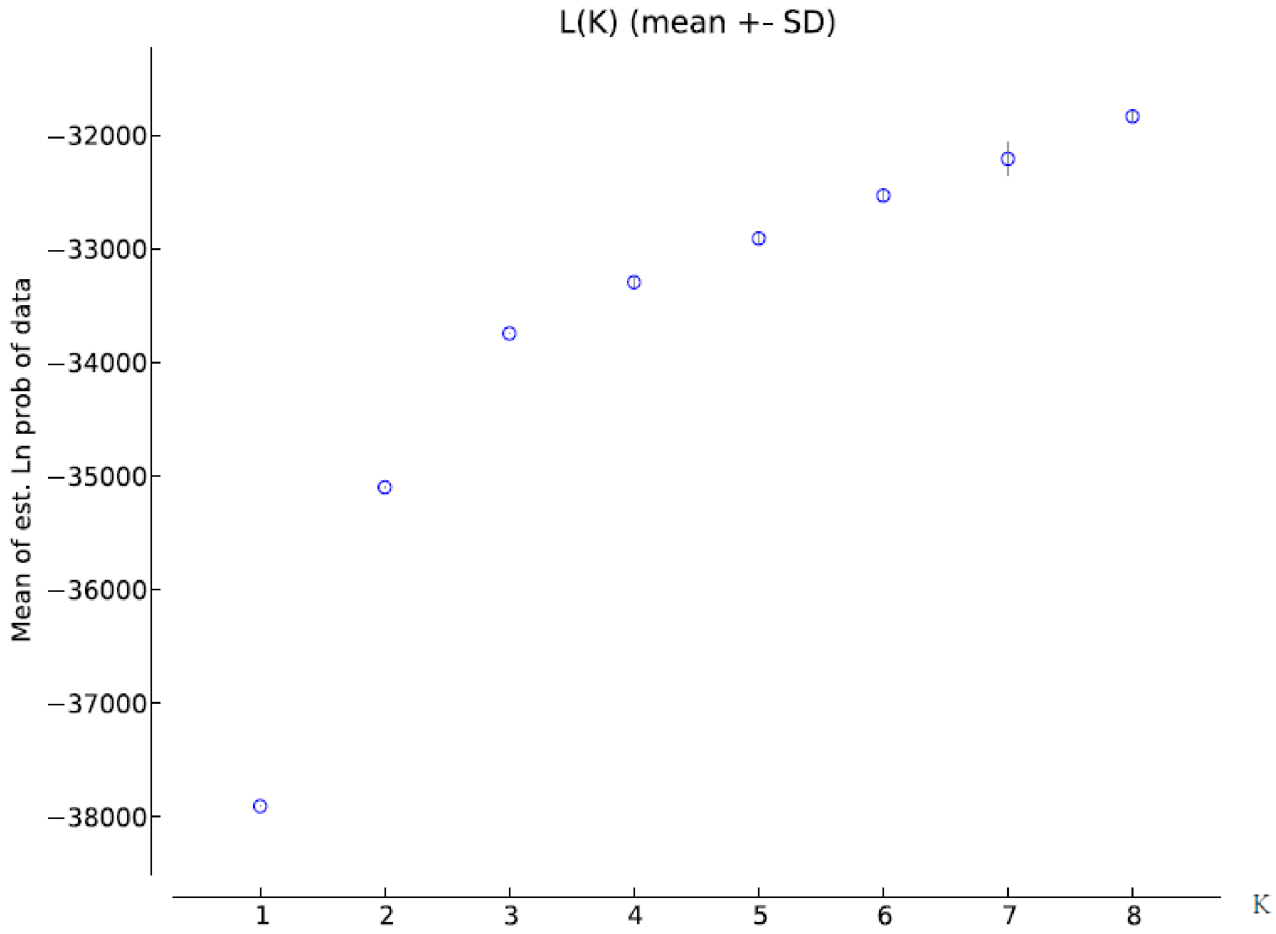

Fig. S1: Estimation of the optimal value of *K* using the L(*K*) method. According to this method, L(*K*) plateaus when *K* approaches the true value; which is *K* = 3 in this case.

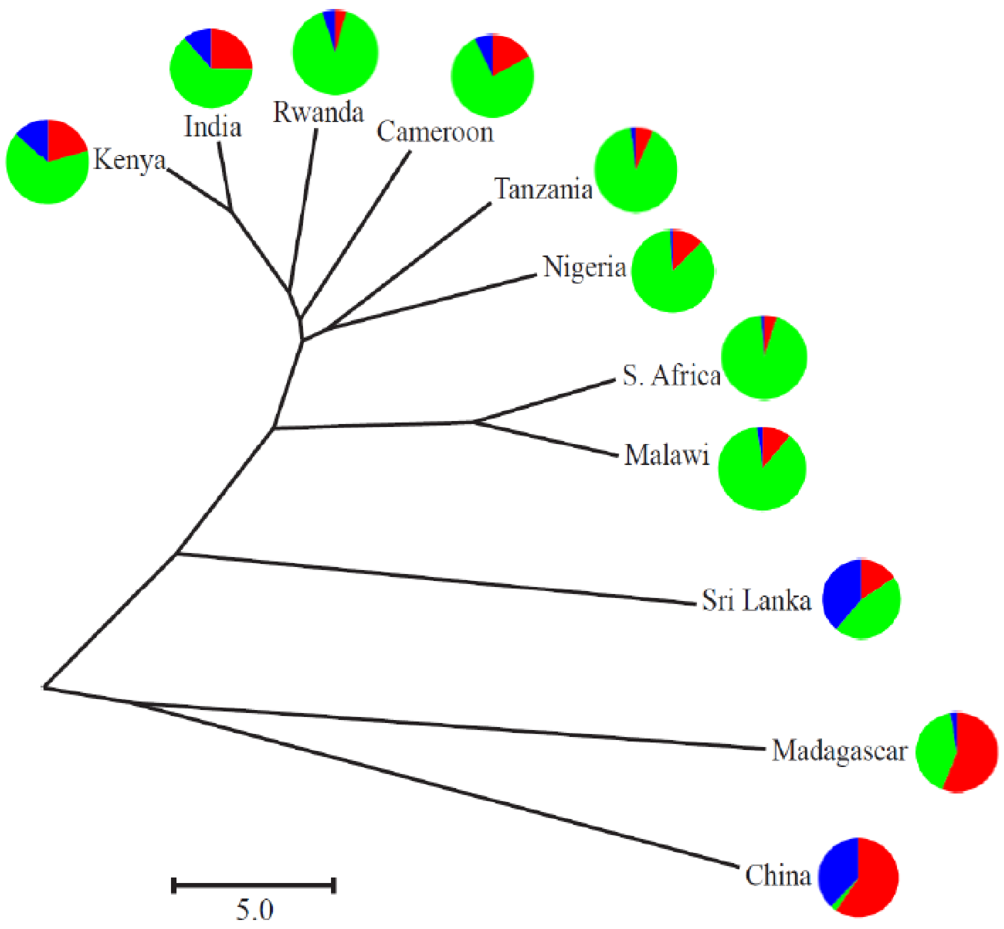

Fig. S2: Unrooted Fitch–Margoliash tree based on Nei's standard genetic distance between tea accessions from 11 countries. The colours correspond to genetic clusters defined by the STRUCTURE analysis at K = 3 in Fig. 1 with Group 1 (China tea) in red, Group 2 (Indian Assam tea) in green and Group 3 (Chinese Assam tea) in blue.

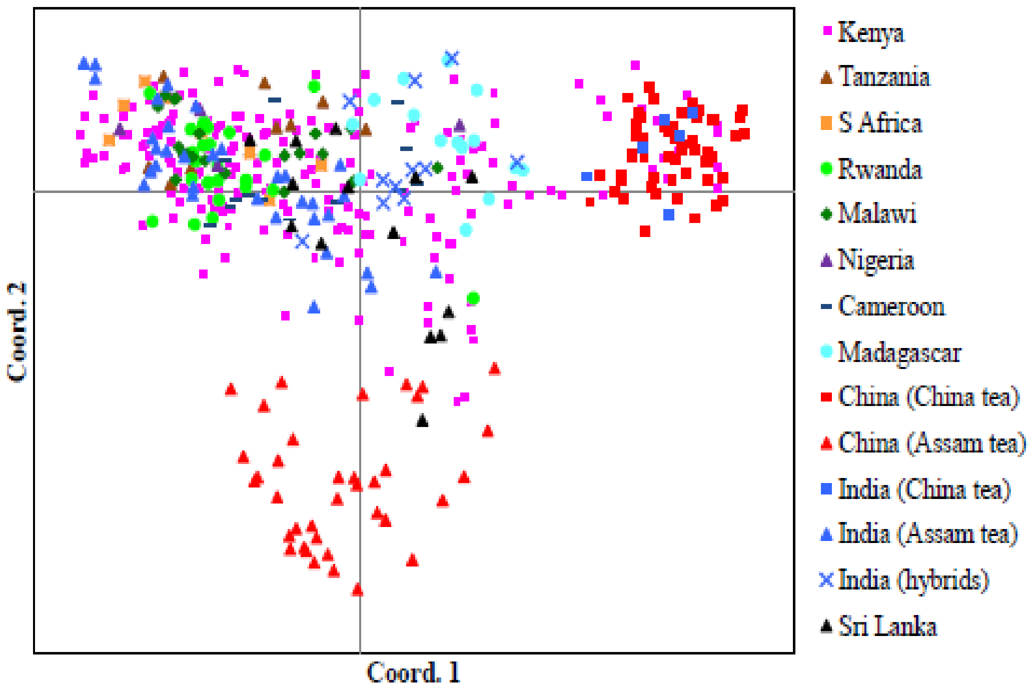

Fig. S3: Principal co-ordinates analysis (PCoA) for African and Asian tea accessions based on 23 nSSR loci. Coord 1 and Coord 2 represent the first and second co-ordinates, respectively. The two co-ordinates accounted for 59.60% of the total variation.

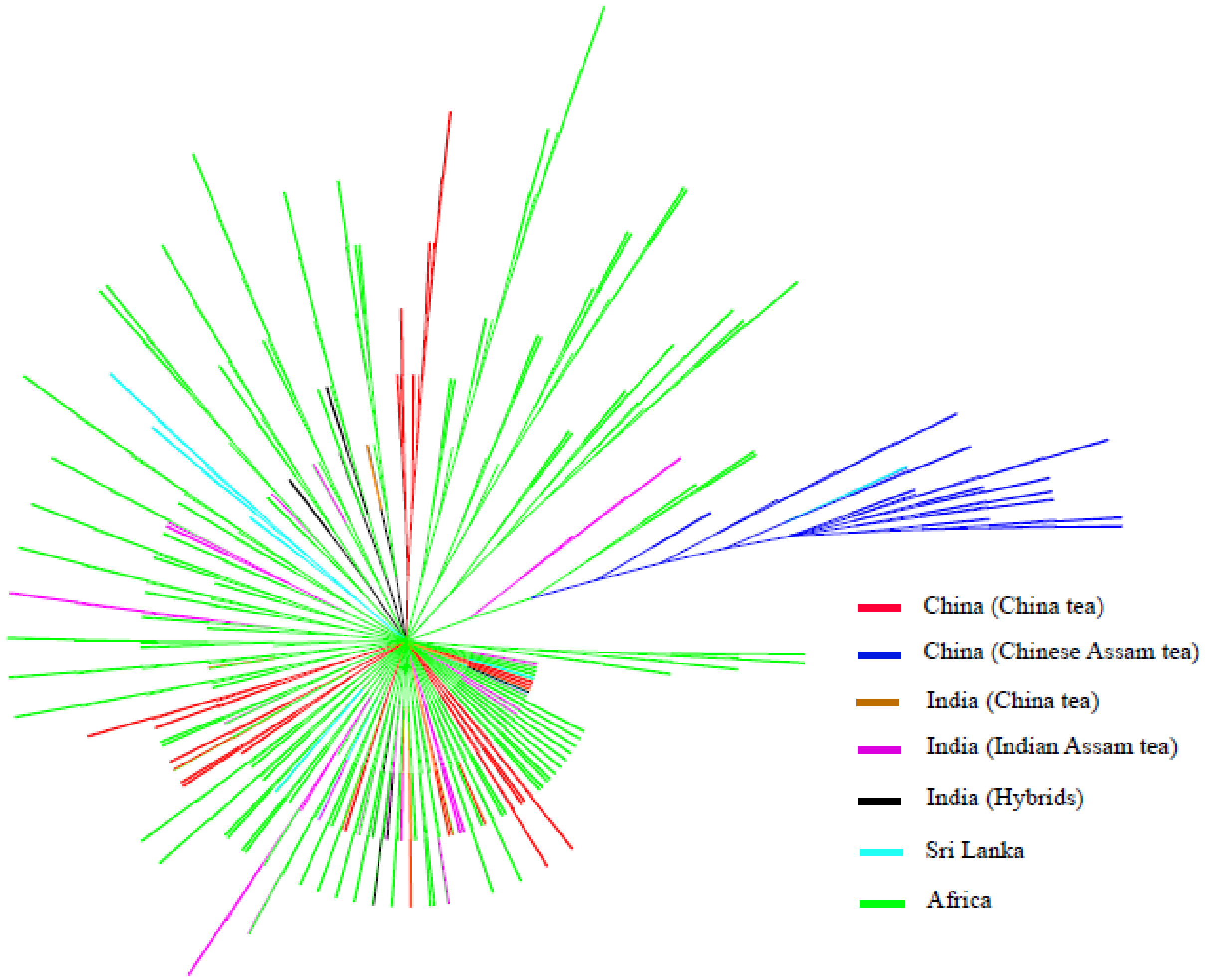

Fig. S4: Neighbour joining tree of 439 tea accessions from 11 countries in Africa and Asia. The tree was constructed from Nei's genetic distances. Different colours represent the various geographic regions as shown in the figure.

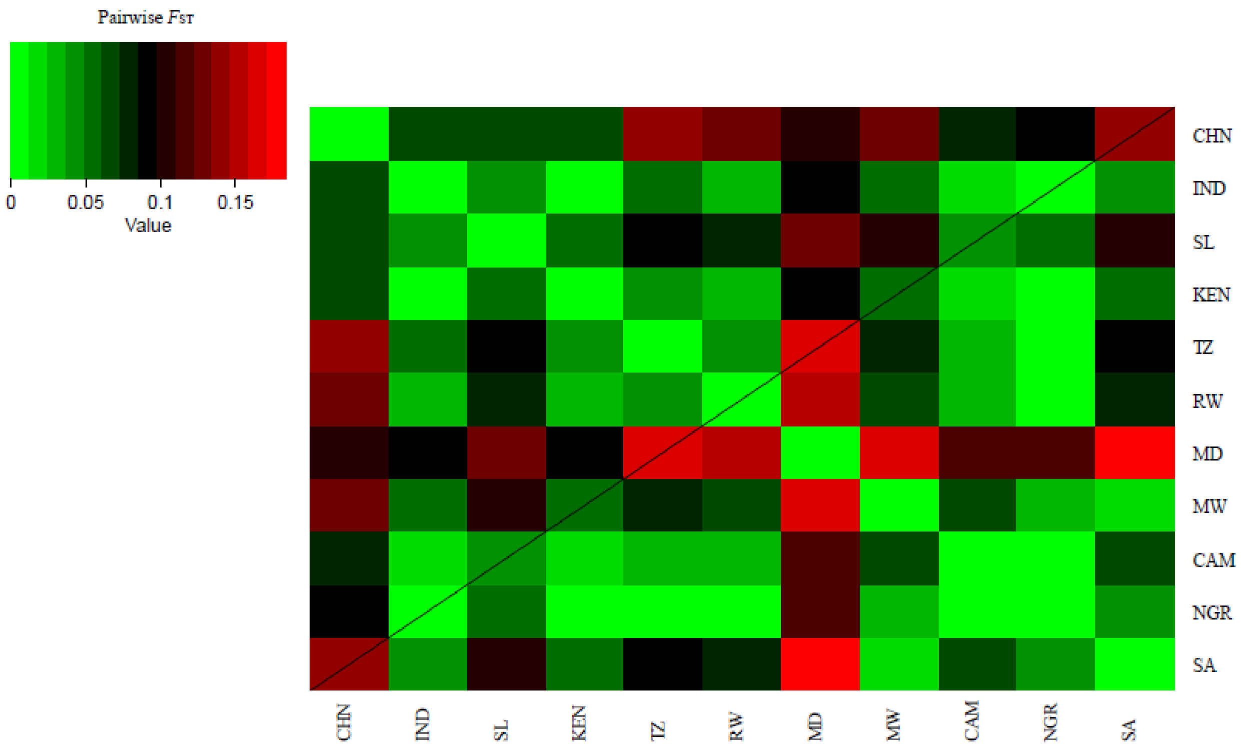

Fig. S5: Heat map view of pairwise FST among countries. The countries are arranged in the order of increasing geographic distance from China to South Africa.

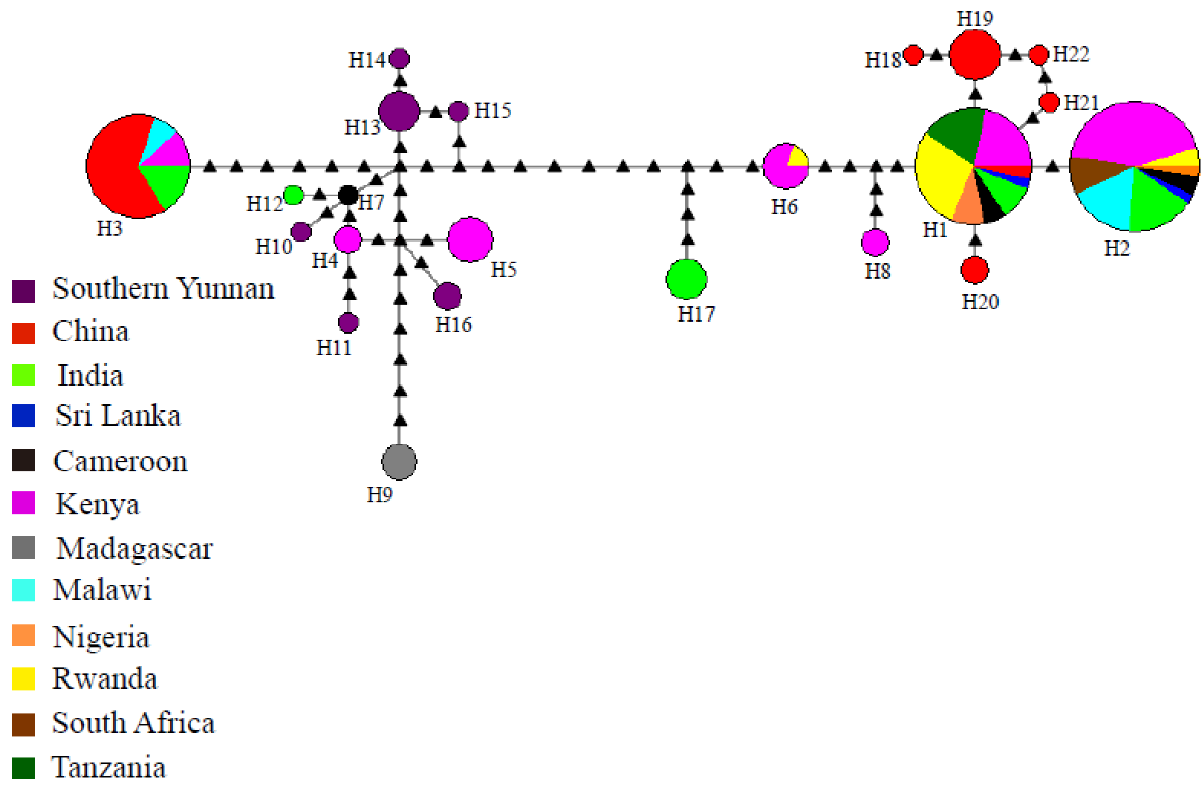

Fig.S6: Median joining network of haplotypes showing the distribution of the haplotypes in the 11 countries. The size of the circles corresponds to the frequency of each haplotype whereas the small black triangles represent mutational steps.

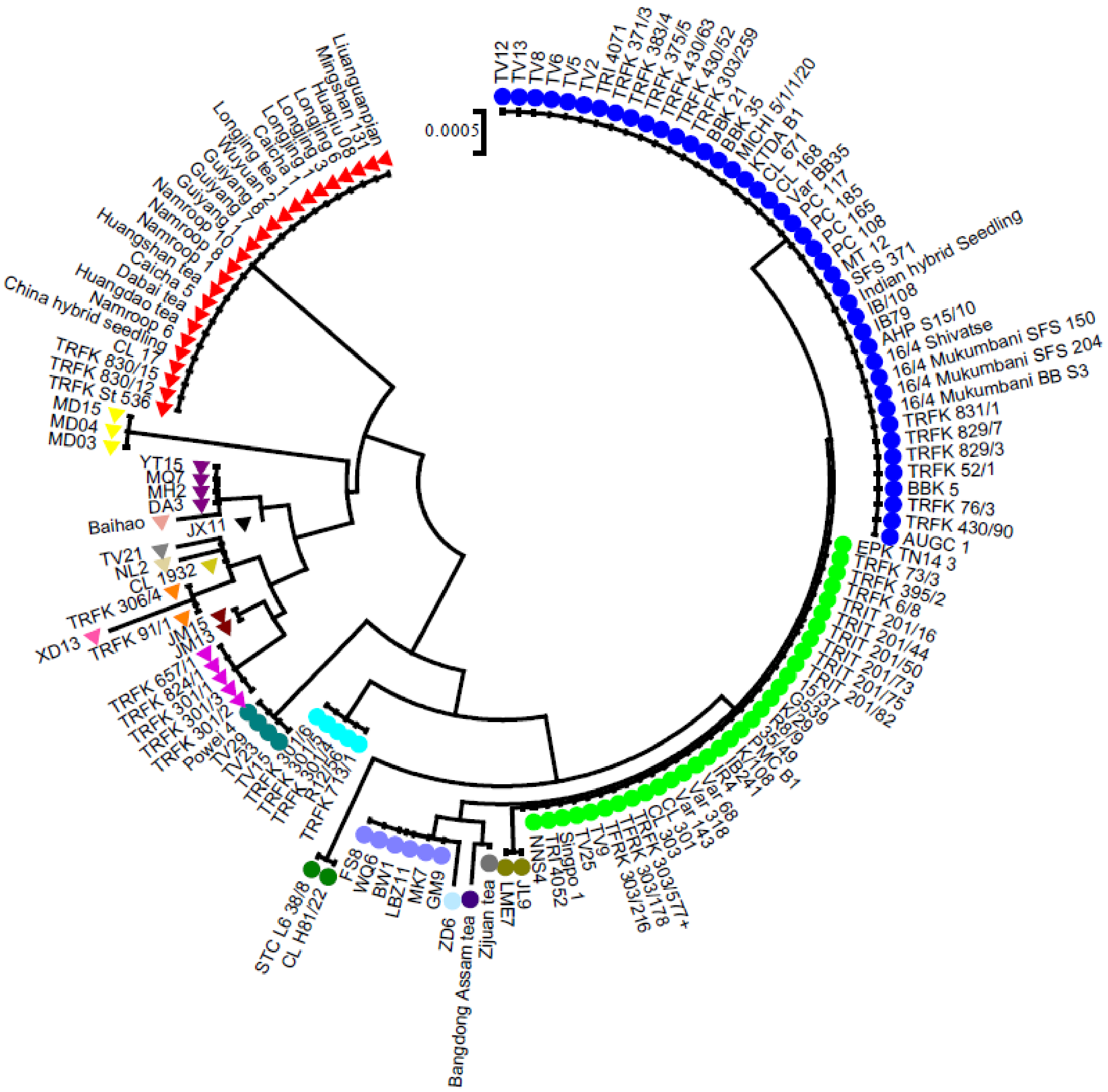

Fig. S7: An unrooted neighbour joining tree of 143 cpDNA sequences of cultivated tea. The tree is drawn to scale, with branch lengths in the same units as those of the evolutionary distances used to infer the tree. The colours correspond to the different haplotypes depicted in **Fig 5**.

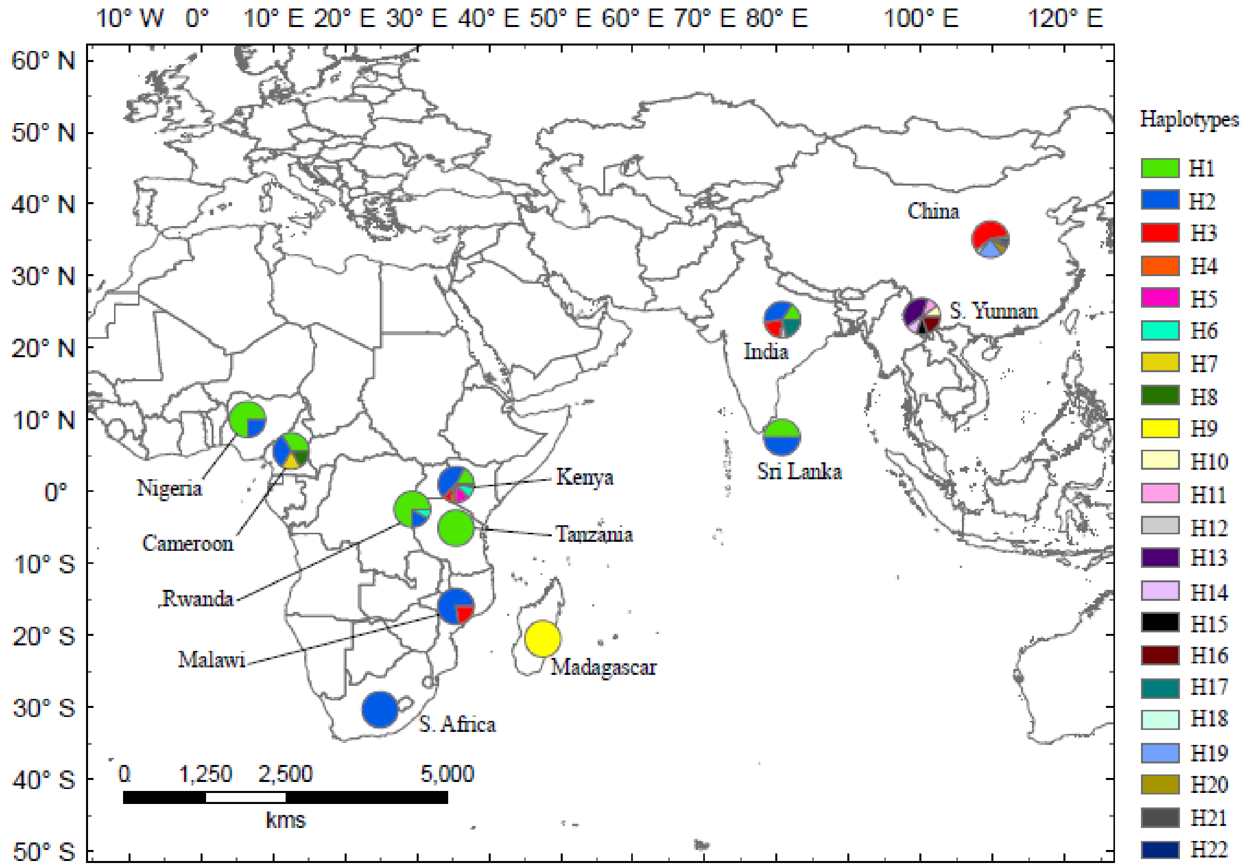

Fig. S8: Distribution of tea cpDNA haplotypes across 11 countries mapped using ArcGIS. The colours represent different haplotypes. The map was generated using DIVA-GIS and ArcGIS software as described for **Figure 2.**
